# Diagnosis and Treatment of Depression in Spain: Are There Gender Inequalities?

**DOI:** 10.3390/ijerph17249232

**Published:** 2020-12-10

**Authors:** Andrea Cabezas-Rodríguez, Amaia Bacigalupe, Unai Martín

**Affiliations:** 1 Department of Sociology and Social Work, University of the Basque Country (UPV/EHU), 48940 Leioa, Spain; amaia.bacigalupe@ehu.eus (A.B.); unai.martin@ehu.eus (U.M.); 2 Doctorate Program in Public Health, Department of Preventive Medicine and Public Health, University of the Basque Country (UPV/EHU), 48940 Leioa, Spain; 3 Social Determinants of Health and Demographic Change—Opik Research Group, University of the Basque Country (UPV/EHU), 48940 Leioa, Spain

**Keywords:** mental health, depression, gender inequalities, medicalisation

## Abstract

It is well known that women are more likely than men to be diagnosed with depression and to consume antidepressants. The factors related to the medicalisation of depression and their social distribution remain unclear. The aim of this study was to analyse gender inequalities in the medicalisation of depression from an intersectional perspective. This was a cross-sectional study based on data from the European Health Survey relating to Spain. Gender inequalities were calculated using prevalence ratios of women compared to men with a diagnosis of depression and antidepressant use, adjusted for age, depressive symptoms, primary care visits and diagnosis of depression in the case of antidepressant use. After adjustments, the diagnosis of depression and the use of antidepressants were more prevalent in women, especially of lower socioeconomic levels. Gender inequalities in the diagnosis of depression also increased with decreasing level of education. Regarding the use of antidepressants, gender inequalities were not significant in university graduates and people of higher social. The gender inequalities found in the diagnosis and treatment of depression cannot be completely attributed to a higher level of depressive symptoms in women or their greater frequency of visits to primary care. Inequalities are greater in more vulnerable social groups.

## 1. Introduction

The higher rates of depression and psychotropic drug prescription recorded among women represent a consistent finding in the international literature [1,2,3]. In Spain, the National Health Survey in 2017 reported the prevalence of depression diagnoses in women to be twice that in men—9.2% compared to 4.0%—while the prescription and consumption of antidepressants and stimulants was also more than double in women (7.9% versus 3.2%) [4].

Several possible explanations for these differences have been put forward. The first is that the higher frequency of diagnoses in women is a consequence of their poorer mental health. On this view, the biological differences between men and women, specifically hormonal differences, have been proposed as a fundamental explanatory factor for the propensity of women to suffer higher levels of mental pathology [5]. However, this biological explanation has been shown to be insufficient [6], and sociological perspectives have suggested that the reason lies in the gender inequalities that can be traced to the social stratification between men and women. This focus posits that the vertical and horizontal segregation of the labour market, the greater participation of women in domestic and care work, and their greater exposure to situations of sexual violence and discrimination [7] have a decisive negative impact on their mental health [8].

Other studies, however, have suggested that poorer mental health is not the sole reason for the frequency of diagnosis of depression in women. They propose that medicalisation—a process through which various spheres of human life have been incorporated into medical discourse and practice [9]—in the area of women’s mental health may have increased the frequency of diagnosis of depression or anxiety due to biases in the diagnostic process. Although research in this field is scarce, results indicate that mental health practitioners diagnose depression and/or anxiety [10,11] and prescribe psychotropic drugs more frequently in women than in men with the same symptoms [12]. These results are attributed to the fact that healthcare professionals are influenced by gender stereotypes and socially accepted definitions of hegemonic female and male characteristics. This social mindset is more permissive of low mood among women, and women tend to express their problems more openly in psychosocial terms; therefore their rate of depression diagnosis is higher [13]. The screening tools themselves and other psychometric instruments for measuring depression may also be subject to gender biases [14], deriving precisely from the fact that normative characteristics of female behaviour such as crying or hypersensitivity are interpreted as symptoms of depression, while other cognitive or affective symptoms more typical of men are not [15]. Likewise, due to the disparities in the socialisation of the sexes, men have more difficulty in expressing their emotions or in accepting the need to seek professional help, and so their rate of depression diagnosis is lower [16]. Finally, because women have more contact with primary care services, symptoms of depression are more likely to be detected in women than in men, resulting in a greater number of diagnoses and prescriptions [17].

In addition to gender, there are other dimensions of social inequality that place women from the most disadvantaged groups at a high risk of suffering from depression and of experiencing gender biases in the treatment they receive. Several studies applying an intersectional approach have reported that women from more disadvantaged social classes [18] or with lower levels of schooling [19] are more frequently diagnosed with depression or anxiety than women of other social or educational status. Likewise, the dominant societal norms regarding gender identity and sexual orientation may increase the likelihood of poor mental health in people who are not cisnormative and/or heterosexual because they do not conform to the expectations imposed by the binary construction of sex [20,21]. Therefore, incorporating intersectionality theory may achieve a more detailed understanding of how the different dimensions of inequality interact in the medicalisation of depression and, in turn, may promote the search for solutions and enhance health equity [22].

In general, the research carried out in this field does not differentiate between these pathologies, and as a result, depression, anxiety, and other types of common mental disorder have tended to be analysed together. This means that it is impossible to trace the process of medicalisation in each disorder independently or to establish whether or not they present distinct characteristics. Another problem is that most of the studies are clinically based [10,11]; very few population-based studies have been carried out that might give an idea of the degree of medicalisation in a particular population. Using data on depressive symptoms alone (i.e., excluding other disorders) on the medical diagnosis of depression and antidepressant use in a specific population, the present study has the means necessary to assess the process of the medicalisation of depression from a gender perspective. Thus, the aim of this article was to analyse gender inequalities in the medicalisation of depression in the Spanish population, taking into account the possible influences of different dimensions of social inequality. We hypothesised that in patients of both sexes with the same depressive symptoms, women would be more frequently diagnosed with depression than men; additionally, that in patients of both sexes with the same number of depression diagnoses, women would be prescribed more antidepressants.

## 2. Materials and Methods

A cross-sectional study was carried out using data corresponding to a Spanish sample from the 2014 European Health Interview Survey (EHIS), coordinated by Eurostat and administered by the Spanish National Statistics Institute. The survey was applied to a randomly selected sample of people (*n* = 22,842) from the non-institutionalised population. The response rate was 74.6%. The analysis carried out here corresponds to the data obtained from 20,313 people aged 25 years and over.

The outcome variables were the medical diagnosis of depression and the consumption of antidepressants and/or stimulants. Both assessments were based on the respondent’s self-reports. Diagnosis of depression was accepted in the case of an affirmative answer to the item “depression” in the list of frequent chronic problems diagnosed by medical staff. In the case of the consumption of psychotropic drugs, the question on medicine use prescribed by a doctor over the last two weeks was used: respondents who marked the item “antidepressants, stimulants” from the list of the most frequent medications were considered to be consuming these drugs.

The variables related to socioeconomic status were the highest level of schooling attained (categorised as primary school, secondary school, or university) and occupational social status (current, or most recent for those not in work) categorised into five groups, in accordance with the guidelines of the Spanish Society of Epidemiology [23].

As adjustment variables, age, presenting depressive symptoms, and the number of visits to primary care were used. Depressive symptomatology was evaluated using the Patient Health Questionnaire (PHQ-8), which is used to detect depressive symptoms in the general population. The PHQ-8 score ranges from 0 to 24 points and is obtained from the sum of the scores of the items; a score of 5 or more is taken to indicate the presence of depressive symptoms. The number of primary care visits in the last four weeks was recorded. In the analysis of the consumption of psychotropic drugs, the medical diagnosis of depression described above was also used as an adjustment variable.

The crude prevalence of the diagnosis of depression and use of psychotropic drugs were calculated for each sex according to age, social status and level of education. To analyse the differences between men and women in the two outcome variables, prevalence ratios (PRs) were calculated using Poisson regression models with robust variance, taking male gender as a reference. Sequential PRs adjusted for the adjustment variables described above were calculated. Thus, in the case of the diagnosis of depression, the differences between men and women were calculated through PRs adjusted for age and subsequently for depressive symptoms and primary care visits. For the use of psychotropic drugs, along with these three variables, the medical diagnosis of depression was included as an added adjustment variable. The sample weight provided by the designers of the survey was used. A more detailed description of the survey methodology, including the treatment of missing data, can be found in the National Statistics Institute’s publication [24]. The analyses were performed with SPSS 25 (IBM, Armonk, NY, USA).

## 3. Results

Table 1 displays the sample’s characteristics. Depressive symptoms were present in 21.5% of women and 11.4% of men. The prevalence of a medical diagnosis of depression was much higher in women (13.5%) than men (5.8%), as was the consumption of prescribed antidepressants or stimulants (8.7% in women and 3.5% in men). Women also made more primary healthcare visits. Regarding sociodemographic characteristics, most of the population were between 25 and 49 years old; most belonged to social class IV and, in general, secondary school was the highest level of education attained. All differences between sexes were statistically significant.

In both men and women, diagnoses of depression and the consumption of prescribed antidepressants and/or stimulants increased with age and decreased with higher social status and level of education (Table 2). Women with the lowest level of education had the highest prevalence of diagnosis (21.2%), a figure far higher, for example, than that of male university graduates (3.2%). The same pattern was observed in the consumption of antidepressants and/or stimulants; they were taken by 13.8% of women with the lowest level of education and by only 2.0% of men with the highest.

In all cases, and after all adjustments, women presented a greater likelihood of being diagnosed with depression and of using psychotropic drugs than men. Gender inequalities (Table 3) in the diagnosis of depression increased with age, but were significant at all ages. In the older group, the adjusted difference of receiving a diagnosis of depression was almost twice as high in women as in men [PR_80+_ = 1.95 (1.42–2.67)], even after adjusting for depressive symptoms and the number of visits to primary care. In the use of antidepressants, no such clear gender inequalities were observed: the differences were only significant in the intermediate age groups [PR_50–64_ = 1.44 (1.19–1.73)] and [PR_65–79_ = 1.28 (1.02–1.62)].

By social class (Table 4), in the diagnosis of depression there was no clear social gradient, but there were gender inequalities in the lower social classes (PR _social class IV_ = 1.62 (1.43–1.83)) and (PR_social class V_ = 2.17 (1.71–2.76)), as well as in social class I. In the case of drug use, clear gender inequalities were observed in social classes III, IV and V.

According to level of education (Table 5), a clear social gradient was seen: as the educational level decreased, gender inequalities increased, both for the diagnosis of depression [PR _primary school_ = 1.74 (1.52–2.00)], and for the consumption of antidepressants [PR _primary school_ = 1.37 (1.16–1.61)].

## 4. Discussion

To the best of the authors’ knowledge, the present study is the first to apply an intersectional perspective to the analysis of gender inequalities in the diagnosis of depression and in the use of antidepressants. The main finding is that depression was diagnosed significantly more often in women than in men, even though they did not present more depressive symptoms or make more visits to primary care services. Likewise, women also consumed more prescription antidepressants and stimulants than men. From an intersectional point of view, gender inequalities in the diagnosis of depression increased with age and were also greater in the most disadvantaged social groups. In low social status groups, there were also notable gender inequalities in the consumption of antidepressants and/or stimulants.

As other studies have indicated [1,2,3] the higher prevalence of the diagnosis of depression and the use of antidepressants in women is probably attributable not to a single cause but to the sum of different factors. However, many studies in this area do not stratify their results by sex [25], therefore it is difficult to identify the causes. Our results are consistent with previous work reporting the existence of a higher rate of diagnoses of depression in women than in men with the same depressive symptoms, as well as a greater consumption of psychotropic drugs [10,11,12,26]. The search for an explanation of these results is complex, because they perhaps derive from a paradoxical process in which women may be over-diagnosed and treated for depression, and men under-diagnosed and treated [27]. From a gender perspective, then, we are witnessing a growth in the pathologisation of daily life stresses which has a clear gender component; frequently, the emotional distress suffered by women due to their social position in society is classified as depressive pathology, and in turn generates a demand for unnecessary treatment [28,29]. Furthermore, the distinct socialisation of emotions in men and women implies that traditionally feminine attributes such as sensitivity, crying and emotional lability favour their identification with the hegemonic definition of depression, which was constructed on the basis of a stigmatisation of women’s identity as weaker subjects who were less resilient to suffering. As a result, it may be that health practitioners are more given to identify symptoms that constitute the classic diagnosis of depression among women [8]. Another possible consequence of this social construction of gender is an underdiagnosis of depression in men. The social expectations of the hegemonic male identity, with its emphasis on the concealment of emotions (especially those classified as female) may increase the difficulty of diagnosing depression in the male population [15]. Likewise, it is important to highlight that societal cisnormativity and heteronormativity has historically generated an added degree of mental suffering, by classifying people who depart from the predominant pattern as mentally ill. Progress has been made in the depathologisation of sexual diversity and in promoting recognition and respect for the diversity of gender expressions and identities [20].

Although evidence of the interaction between gender and other dimensions of social inequality in the medicalisation of depression is currently lacking, research incorporating intersectionality theory in other areas of health highlights its importance; the inequalities identified when considering several dimensions together are greater than when these dimensions are considered separately [30,31]. Our results indicate that belonging to the most vulnerable social groups increases gender inequalities in the diagnosis of depression and in the treatment prescribed. Similarly, the results of another study [32] about the medicalisation of anxiety and depression, pointed to the over-medicalisation of mental health in women and confirmed the importance of an intersectional approach; that study found that the most vulnerable social groups were more likely to suffer from the medicalisation of their mental health than their peers, and reported an even clearer social gradient than the one observed here. This suggests that the interrelationship between different dimensions of inequality may influence the response of practitioners to women of lower socioeconomic status and may thus increase the latter’s vulnerability to gender biases. Indeed, one systematic review found that less educated, lower-income patients received less diagnostic information from their physicians, who adopted a less participatory consulting style and thus restricted the role of the patients in decision-making regarding their treatment [33]. Another study conducted with primary care physicians on the diagnosis of coronary heart disease showed that, with respect to men, women were asked fewer questions and underwent fewer physical examinations and diagnostic tests, a practice that may negatively affect the accuracy of the diagnosis and the appropriateness of treatment [34]. The influence of a patient’s gender on the patient–doctor relationship and on the doctor’s decisions regarding treatment has also been shown [35]. In addition to the inequalities in doctor–patient relationships, the greater gender inequality in the medicalisation of depression in the most disadvantaged groups may be due to the greater presence of traditional male values among low socioeconomic status men [36], which may lead to a neglect of symptoms and in turn to undertreatment. In the case of women of lower social status, the need to resume their daily family responsibilities may foster a proactive attitude to requesting medication in order to make a speedy recovery. However, more research from an intersectional perspective is needed to better understand the mechanisms underlying these results.

This study presents the characteristic limitations of survey-based cross-sectional studies. Firstly, its use of self-reported data obtained from a health survey may not coincide with the data obtained through the use of clinical databases [37]. However, population health surveys are currently the tools most widely used to determine the prevalence of clinical problems at the population level. In addition, using data from health surveys allows information to be obtained from the entire population and not just from those who use the public health system, as is the case with the digital medical records in our setting. Health surveys offer the added advantage of containing a multitude of socioeconomic data that are not available in health records. Another important limitation is the possible gender bias present in the instrument for assessing depressive symptoms, because most of the tools used for this purpose use criteria that classify characteristics traditionally understood as female as depressive symptoms [15]. The PHQ-8 used in this study may underestimate the number of men with depressive symptoms. However, if this were the case, it would underestimate gender inequalities in the medicalisation of mental health, and this would actually reinforce our findings. In any case, the PHQ-8 is a widely used and validated instrument for measuring depressive symptoms in the general population.

The medicalisation of mental health has direct consequences for women’s health, due either to pharmacologic iatrogenesis or to the development of both physical and psychological dependence on psychoactive drugs and health professionals. In addition, the medicalisation of daily ailments makes it difficult to explore the structural roots of gender inequalities in mental health, because it individualises collective social problems through personal diagnoses and treatments [13]. Thus, the results of our study have important implications for policy and clinical practice. In the domain of public health, there is evidence of the impact of gender inequality on mental health [38]. Public interventions in the labour market or in the domestic and care settings aiming to reduce this impact will help to narrow the gender gap in mental health as in other areas [39]. Additionally, in the context of clinical practice, our results suggest the need to reconsider the criteria used to create diagnostic categories and treatment guidelines in the current healthcare model. The incorporation of the gender perspective in the attention to the symptoms reported by patients may represent a radical change in the concept of depression and in its treatment, and may give a new meaning to mental suffering. Thus, with the aims of avoiding the pathologisation of distress caused by social circumstances and of steering the current situation of inequality towards a more equitable approach to clinical care, several strategies have emerged as means of protecting against the excessive medicalisation of women’s mental health, including the indication of no-treatment [40], the incorporation of the biopsychosocial model to acquire a global vision of the patient [41], or the incorporation of feminist approaches to narrative psychotherapies [42]. In this way, adopting a holistic approach to the care of the patient, in place of the overriding focus on symptoms currently in vogue, might prompt a less discriminatory attitude to the care of depression which would ultimately reduce the degree of medicalisation of mental health, especially among women.

## 5. Conclusions

Our results show that the diagnoses of depression and the consumption of prescribed antidepressants are more frequent in women than in men. This is the case even though women do not present more depressive symptoms or make more visits to primary care services. Furthermore, gender inequalities in the medicalisation of depression are greater among more vulnerable social groups. These results should be taken into account in the design of interventions aimed at reducing gender inequalities in mental health, and underline the need to incorporate gender perspective in clinical practice.

## Figures and Tables

**Table 1:** Distribution of sample (%) according to sociodemographic, health and socioeconomic variables by sex. European Health Interview Survey (Spain), 2014.

	Men (*n* = 9849)	Women (*n* = 10,464)	*p* (95%)
**Age**			
25–49	52.8	48.8	<0.001
50–64	25.9	25.1	
65–79	15.7	17.4	
80 and over	5.6	8.7	
**Depressive symptoms**	11.4	21.5	<0.001
**Diagnosis of depression**	5.8	13.5	<0.001
**Antidepressants, stimulants consumption**	3.5	8.7	<0.001
**Visits to Primary Care**			
None	73.9	65.6	<0.001
One	20.9	27.1	
Two or more	5.2	7.3	
**Social class**			
I	11.7	11.1	<0.001
II	8.1	8.6	
III	19.2	19.2	
IV	48.2	45.9	
V	12.8	15.2	
**Educational level**			
University	19.0	21.5	<0.001
Secondary	51.8	43.2	
Primary or lower	29.2	35.3	

Source: Created using data extracted from the European Health Interview Survey (EHIS) (Spain), 2014.

**Table 2:** Prevalence (%) of diagnosis of depression and antidepressants/stimulants consumption in the last two weeks according to age, social class and educational level in men and women. European Health Interview Survey (Spain), 2014.

	Diagnosis of Depression (%)		Antidepressants, Stimulants Consumption (%)	
	Men	Women	Men	Women
**Age**				
25–49	3.7	7.5	2.4	4.6
50–64	7.9	17.3	4.4	11.9
65–79	7.8	21.7	5.0	13.8
80 and over	9.0	20.5	6.1	12.9
**Social class**				
I	2.8	6.5	2.7	4.2
II	5.6	8.7	3.2	5.2
III	5.6	9.8	3.2	6.6
IV	6.4	15.6	3.8	10.1
V	6.3	19.7	3.8	12.6
**Educational level**				
University	3.2	4.9	2.0	3.3
Secondary	5.4	11.6	3.2	7.3
Primary or lower	8.0	21.2	5.0	13.8

Source: Created using data extracted from EHIS (Spain), 2014.

**Table 3:** Prevalence ratio (PR) (IC 95%) of diagnosis of depression and antidepressant/stimulant consumption by age according to different adjustments ^1^ (reference category: men). European Health Interview Survey (Spain), 2014.

	Model 1	Model 2	Model 3	Model 4
**Diagnosis of depression**				
25–49	2.05 (1.73–2.43)	1.60 (1.35–1.88)		1.50 (1.27–1.77)
50–64	2.17 (1.86–2.54)	1.55 (1.34–1.80)		1.51 (1.30–1.75)
65–79	2.57 (2.12–3.12)	1.68 (1.39–2.03)		1.70 (1.40–2.05)
80 and over	2.72 (1.98–3.74)	1.95 (1.42–2.67)		1.95 (1.42–2.67)
**Antidepressants/stimulants consumption**				
25–49	1.91 (1.55–2.36)	1.43 (1.16–1.76)	1.20 (1.00–1.45)	1.16 (0.96–1.39)
50–64	2.71 (2.19–3.35)	1.91 (1.55–2.35)	1.48 (1.22–1.78)	1.44 (1.19–1.73)
65–79	2.58 (2.01–3.30)	1.71 (1.33–2.20)	1.28 (1.02–1.62)	1.28 (1.02–1.62)
80 and over	2.55 (1.73–3.75)	1.93 (1.29–2.89)	1.41 (0.98–2.02)	1.40 (0.98–2.01)

^1^ Model 1: Crude; Model 2: Adjusted by depressive symptoms; Model 3: Adjusted by depressive symptoms and diagnosis of depression; Model 4: Adjusted by depressive symptoms and number of visits to Primary Care in the case of the variable “Diagnosis of depression”; and adjusted by depressive symptoms, diagnosis of depression and number of visits to Primary Care in the case of the variable “Antidepressants/stimulants consumption”. Created using data extracted from EHIS (Spain), 2014.

**Table 4:** PR (IC 95%) of diagnosis of depression and antidepressants/stimulants consumption by social class according to different adjustments ^1^ (reference category: men). European Health Interview Survey (Spain), 2014.

	Model 1	Model 2	Model 3	Model 4
**Diagnosis of depression**				
I	2.13 (1.42–3.21)	1.80 (1.20–2.69)		1.84 (1.22–2.77)
II	1.63 (1.13–2.34)	1.32 (0.93–1.87)		1.28 (0.91–1.80)
III	1.63 (1.29–2.05)	1.17 (0.93–1.47)		1.16 (0.92–1.45)
IV	2.36 (2.07–2.68)	1.64 (1.45–1.86)		1.62 (1.43–1.83)
V	2.97 (2.33–3.77)	2.24 (1.76–2.84)		2.17 (1.71–2.76)
**Antidepressants/stimulants consumption**				
I	1.45 (0.93–2.28)	1.23 (0.80–1.90)	0.82 (0.55–1.21)	0.78 (0.53–1.14)
II	1.71 (1.04–2.82)	1.41 (0.86–2.32)	1.20 (0.74–1.93)	1.18 (0.73–1.90)
III	2.11 (1.55–2.87)	1.59 (1.15–2.19)	1.53 (1.16–2.01)	1.55 (1.18–2.04)
IV	2.48 (2.10–2.93)	1.69 (1.43–2.00)	1.33 (1.15–1.55)	1.33 (1.14–1.54)
V	3.05 (2.24–4.17)	2.14 (1.57–2.92)	1.39 (1.05–1.83)	1.38 (1.05–1.82)

^1^ Model 1: Adjusted by age; Model 2: Adjusted by age and depressive symptoms; Model 3: Adjusted by age, depressive symptoms and diagnosis of depression; Model 4: Adjusted by age, depressive symptoms and number of visits to Primary Care in the case of the variable “Diagnosis of depression”; and adjusted by age, depressive symptoms, diagnosis of depression and number of visits to Primary Care in the case of the variable “Antidepressants/stimulants consumption”. Created using data extracted from EHIS (Spain), 2014.

**Table 5:** PR (IC 95%) of diagnosis of depression and antidepressants/stimulants consumption by educational level according to different adjustments ^1^ (reference category: men). European Health Interview Survey (Spain), 2014.

	Model 1	Model 2	Model 3	Model 4
**Diagnosis of depression**				
University	1.86 (1.35–2.56)	1.54 (1.12–2.11)		1.52 (1.10–2.10)
Secondary	2.08 (1.81–2.39)	1.58 (1.38–1.81)		1.54 (1.35–1.77)
Primary or lower	2.59 (2.25–2.98)	1.75 (1.53–2.01)		1.74 (1.52–2.00)
**Antidepressants/stimulants consumption**				
University	2.06 (1.37–3.10)	1.65 (1.10–2.48)	1.20 (0.84–1.72)	1.20 (0.84–1.72)
Secondary	2.14 (1.78–2.57)	1.59 (1.33–1.90)	1.30 (1.10–1.52)	1.27 (1.08–1.49)
Primary or lower	2.70 (2.25–3.23)	1.83 (1.52–2.20)	1.37 (1.16–1.61)	1.37 (1.16–1.61)

^1^ Model 1: Adjusted by age; Model 2: Adjusted by age and depressive symptoms; Model 3: Adjusted by age, depressive symptoms and diagnosis of depression; Model 4: Adjusted by age, depressive symptoms and number of visits to Primary Care in the case of the variable “Diagnosis of depression”; and adjusted by age, depressive symptoms, diagnosis of depression and number of visits to Primary Care in the case of the variable “Antidepressants/stimulants consumption”. Authors’ own elaboration from EHIS (Spain), 2014.
